# Chromosomes of parasitic wasps of the superfamily Chalcidoidea (Hymenoptera): An overview

**DOI:** 10.3897/CompCytogen.v14i3.56535

**Published:** 2020-08-25

**Authors:** Vladimir E. Gokhman

**Affiliations:** 1 Botanical Garden, Moscow State University, Moscow 119234, Russia Moscow State University Moscow Russia

**Keywords:** base-specific fluorochromes, chalcid wasps, differential staining, FISH, karyotypes, phylogeny, taxonomy

## Abstract

An overview of the current knowledge of chromosome sets of the parasitoid superfamily Chalcidoidea is given. Karyotypes of approximately 240 members of this group, i.e. just above one percent of described species, are studied up to now. Techniques for obtaining and analyzing preparations of chalcid chromosomes are outlined, including the so-called “traditional” and “modern” methods of differential staining as well as fluorescence in situ hybridization (FISH). Among the Chalcidoidea, the haploid chromosome number can vary from n = 3 to n = 11, with a clear mode at n = 6 and a second local maximum at n = 10. In this group, most chromosomes are either metacentric or submetacentric, but acrocentrics and/or subtelocentrics also can predominate, especially within karyotypes of certain Chalcidoidea with higher chromosome numbers. The following main types of chromosomal mutations are characteristic of chalcid karyotypes: inversions, fusions, translocations, polyploidy, aneuploidy and B chromosome variation. Although karyotype evolution of this superfamily was mainly studied using phylogenetic reconstructions based on morphological and/or molecular characters, chromosomal synapomorphies of certain groups were also revealed. Taxonomic implications of karyotypic features of the Chalcidoidea are apparently the most important at the species level, especially among cryptic taxa.

## Introduction

The superfamily Chalcidoidea is a very diverse, taxonomically complicated and economically important group of insects (Quicke 1997; Gokhman 2015b) that currently includes about 23 thousand described species (Huber 2017). Nevertheless, chromosomes of approximately 240 members of this group, i.e. just above one percent, are studied up to know (Gokhman 2009 onwards). The last detailed review of the chromosome study of Chalcidoidea was published more than a decade ago (Gokhman 2009, see also Gokhman and Gumovsky 2009), with only about 170 examined species. Consequently, important results of the karyotypic study of chalcids accumulated during this time, especially those obtained with the help of certain advanced techniques, substantially changed our views on the phylogenetic and taxonomic implications of chromosomal characters of this group (Gokhman 2013; Baur et al. 2014; König et al. 2019). An updated overview of the karyotypic study of the superfamily Chalcidoidea is therefore given below.

## Techniques used for the chromosome study

Perhaps it is needless to mention that tissues with relatively large numbers of cell divisions should be examined to perform a successful chromosomal analysis of any given group. In the case of Hymenoptera, this for a long time meant studying immature stages (Crozier 1975; Imai et al. 1988; Gokhman 2009). Indeed, chromosome preparations made either from cerebral ganglia or from developing gonads of hymenopteran prepupae and early pupae apparently remain the best source of high-quality metaphase plates, which are the most suitable for morphometric analysis and application of advanced techniques of chromosome staining (Gokhman and Gumovsky 2009). However, obtaining that kind of preparation from many parasitic wasps, including chalcids, is impossible because the establishment of both host and parasitoid lab stocks is usually needed to get access to immature stages of parasitic wasps as well as to ensure reliable identification of this material based on a thorough morphological study of conspecific adults. Nevertheless, this limitation can be overcome in the case of gregarious species (Gokhman 2009). Ovaries of adult females of many parasitoid Hymenoptera can also provide certain numbers of mitotic divisions with discernible morphology of chromosomes, but this mainly applies to synovigenic species, in which oogonia generally continue to divide after eclosion of the female parasitoid from the host (Jervis et al. 2001). On the other hand, ovaries of chalcid wasps often contain meiotic divisions as well, although the number of these divisions is fairly low (Gokhman 2009). In addition, hymenopteran males, which are usually haploid, lack normal meiosis, including synapsis and the reductional division (Crozier 1975), and therefore many details of this process which are observed in diplo-diploid organisms, cannot be reported for parasitoid Hymenoptera. At present, examination of meiotic chromosomes is relatively scarce in Chalcidoidea (see e.g. Gokhman et al. 2014b), but, for example, it would be of considerable interest for studying hybrids between closely related forms with different karyotypes.

Nowadays, the technique developed by Imai et al. (1988) for obtaining air-drying chromosome preparations from prepupae and early pupae of ants, is generally used for karyotyping chalcids. However, stronger hypotonic treatment is usually needed to prevent overlapping of substantially longer chromosomes in the Chalcidoidea. In particular, I normally use 30 min incubation in the 0.5% sodium citrate solution before preparing cell suspension (e.g. Gokhman et al. 2017a), as opposed to 20 min treatment with the 1% solution recommended by Imai et al. (1988). The process also includes maceration of the tissue on the microscope slide in an aqueous solution containing both ethanol and acetic acid, and a subsequent treatment of the cells attached to the slide with an analogous although water-free fixative. However, the final step of chromosome preparation according to Imai et al. (1988), i.e. application of pure acetic acid as an additional fixative, is usually omitted in the case of Chalcidoidea and other parasitoids. I do not only consider this step redundant, but also suggest that the excessive amount of acids can hydrolyze DNA, which is crucial e.g. for performing fluorescence in situ hybridization (FISH). Nevertheless, to avoid washing the cells away from the slide during the subsequent treatment, post-fixation of the material, preferably by acid-free fixatives, is recommended (Gokhman et al. 2019a).

To visualize chromosomes of Chalcidoidea, modern optic microscopes are currently used. Additional epifluorescence modules are also needed to work with fluorochromes, including base-specific chromosome staining and FISH. Moreover, the resulting images must be captured by a modern digital camera, usually controlled through a computer. This camera should produce images with relatively high resolution (at least 300 dpi) and be sensitive enough to work with fluorescence. In turn, these images can be analyzed using specialized software, e.g. KaryoType (Altinordu et al. 2016), to determine absolute/relative lengths and centromere indices of particular chromosomes. As in all other Hymenoptera, chromosomes of chalcid wasps are monocentric, i.e. each of them carries a single centromere (Gokhman 2009). These chromosomes can be subdivided into four groups according to the centromere position, i.e., metacentrics (M), submetacentrics (SM), subtelocentrics (ST) and acrocentrics (A) generally following guidelines provided by Levan et al. (1964). In case of various types of differential staining, both localization and size of particular chromosomal segments have to be identified as well.

It is also noteworthy that precise species identifications are crucial for the karyotypic study of Chalcidoidea as well as of parasitoid Hymenoptera in general (Gokhman 2009). Bearing in mind an exceptional taxonomic complexity of this superfamily and the abundance of cryptic taxa (Gokhman 2018), expert identifications of the examined populations/strains and particular specimens should be obtained in every possible case.

Karyotypes of the overwhelming majority of chalcids were studied using only routine staining. Nowadays, chromosomes of Chalcidoidea are most often stained with Giemsa solution diluted in Sorensen’s phosphate buffer (Gokhman 2009). Nevertheless, routinely stained karyotypes can be further studied using morphometric analysis which already proved its effectiveness for finding both similarities and differences between closely related forms of Chalcidoidea (Gokhman and Westendorff 2000; König et al. 2019). Use of this technique in chalcids is facilitated by the generally low chromosome numbers that are characteristic of most Chalcidoidea.

In addition, karyotypes of a few dozen members of the superfamily Chalcidoidea were examined using various methods of differential staining (Gokhman 2009). The latter techniques are often subdivided into the so-called “traditional” and “modern” ones (Gokhman 2015a). Among the former methods, various techniques of chromosome banding, i.e. C-, AgNOR- and sometimes also G-banding, are used. C- and AgNOR-banding respectively visualize constitutive heterochromatin and nucleolus organizing regions (NORs) (Sumner 1972; Howell and Black 1980). However, chromosomes of only few members of the superfamily Chalcidoidea were studied using either AgNOR- or C-banding. These species belong to the families Aphelinidae (Odierna et al. 1993; Baldanza et al. 1999; Baldanza and Giorgini 2001; Giorgini and Baldanza 2004), Eulophidae (Maffei et al. 2001; Gebiola et al. 2012), Pteromalidae (Reed 1993; Gokhman and Westendorff 2000) and Trichogrammatidae (Van Vugt et al. 2005). C-banding usually visualizes small to medium-sized pericentromeric and telomeric segments of the constitutive heterochromatin on chalcid chromosomes, but a few intercalary blocks were also revealed (Reed 1993; Baldanza et al. 1999; Gokhman and Westendorff 2000). As for AgNOR-banding, it most often detects a single NOR per haploid karyotype (Baldanza et al. 1999; Baldanza and Giorgini 2001 etc.), but two sites of this kind (and an additional NOR on a particular B chromosome) were visualized in the chromosome set of *Trichogrammakaykai* Pinto & Stouthamer, 1997 (Van Vugt et al. 2005). In the superfamily Chalcidoidea, subtelocentric/acrocentric chromosomes usually carry subterminal/terminal NORs, but these sites can be situated close to the centromeres of certain metacentrics (Baldanza et al. 1999; Giorgini and Baldanza 2004). The localization of NORs can vary among members of the same genus (Giorgini and Baldanza 2004), and this is further corroborated by FISH (see below).

G-banding is usually produced by treatment of chromosomes with certain proteolytic enzymes like trypsin (Chiarelli et al. 1972 onwards). Among chalcids, karyotypes of only three members of this group, i.e. *Encarsiaberlesei* (Howard, 1906) and *E.inaron* (Walker, 1839) (Aphelinidae) as well as *Nasoniavitripennis* (Walker, 1836) (Pteromalidae) (Odierna et al. 1993; Baldanza et al. 1999; Rütten et al. 2004) were studied using G-banding. This technique identifies different chromosomes within karyotypes of the same species (Gadau et al. 2015), but apparently fails to highlight homologous elements among chromosome sets of closely related parasitoids (see e.g. Odierna et al. 1993; Baldanza et al. 1999), and therefore it cannot be used for a comparative cytogenetic study of parasitoid Hymenoptera.

The modern techniques of differential chromosome staining are mostly represented by using fluorochromes which specifically visualize AT- and GC-rich chromosome segments (Schweizer and Ambros 1994; Gokhman 2015a). Among the former dyes, 4’, 6-diamidino-2-phenylindole (DAPI) is the most widely used. However, chromosomes of parasitoid Hymenoptera predominantly contain AT-rich DNA, and therefore staining chalcid karyotypes with DAPI and similar fluorochromes normally does not reveal any banding pattern (Odierna et al. 1993; Baldanza et al. 1999 etc.), sometimes except for a single negative band per haploid karyotype (Bolsheva et al. 2012). In turn, bands of this kind, which represent NORs, are usually GC-rich, and thus can be stained with chromomycin A_3_ (CMA_3_) or similar fluorochromes (Gokhman et al. 2019b). Nevertheless, multiple CMA_3_-positive and DAPI-negative terminal bands were recently discovered on every chromosome of a particular member of the family Eulophidae, *Trichospilusdiatraeae* Cherian & Margabandhu, 1942, although it seems unlikely that they all represent NORs (Gokhman et al. 2017b). In addition, there are also several fluorochromes, like propidium iodide, which stain total DNA irrespective of its base composition (Bolsheva et al. 2012).

Nevertheless, FISH remains the most powerful tool for analyzing chromosomes of parasitoid Hymenoptera including chalcids (Gokhman 2015a). This technique seems to work particularly well with different DNA repeats (Van Vugt et al. 2005, 2009). Indeed, it is most frequently used, for example, to map clusters of ribosomal DNA (= NORs) in certain members of Chalcidoidea that belong to the families Eurytomidae, Torymidae, Eulophidae, Aphelinidae and Trichogrammatidae (Van Vugt et al. 2005, 2009; Bolsheva et al. 2012; Gokhman et al. 2014a, 2017a). Among other results, these data show that the number and localization of NORs vary within certain chalcid genera, e.g. *Eurytoma* Illiger, 1807 (Gokhman et al. 2014a; see above). Van Vugt et al. (2005, 2009) also mapped the whole fraction of repetitive DNA (C_0_t-50) as well as the ITS2 and *Eco*RI repeats on chromosomes of *Trichogrammakaykai*. Analogously, Li et al. (2017) used the same approach to physically map a number of repeats on a particular B chromosome of *Nasoniavitripennis*. In addition, FISH revealed absence of the TTAGG telomeric repeat in all studied parasitoid Hymenoptera including chalcids (Gokhman et al. 2014a). Moreover, chromosome microdissection together with whole chromosome painting, a powerful technique for identifying particular chromosomes and their segments, was first applied to the karyotype of *N.vitripennis* more than 15 years ago (Rütten et al. 2004; Gadau et al. 2015). To prepare specific probes from each chromosome of this species which haploid karyotype contains five metacentrics of similar size, the chromosomes were first G-banded. Furthermore, Gokhman et al. (2019a) who applied the same technique to the chromosome sets of two cryptic species of the *Lariophagusdistinguendus* (Förster, 1841) complex (Pteromalidae), were able to identify elements involved in a certain chromosomal fusion (see below).

Methods of immunocytochemistry also can be used for studying karyotypes of parasitoid Hymenoptera. Up to now, however, this technique was applied only to two closely related species, *Entedoncioni* Thomson, 1878 and *E.cionobius* Thomson, 1878 (Eulophidae) (Bolsheva et al. 2012). Specifically, chromosomes of these parasitoids were treated with antibodies against 5-methylcytosine, which visualized patterns of DNA methylation along different chromosomes.

## Overview of known data

### General notes

**Figure 1.**
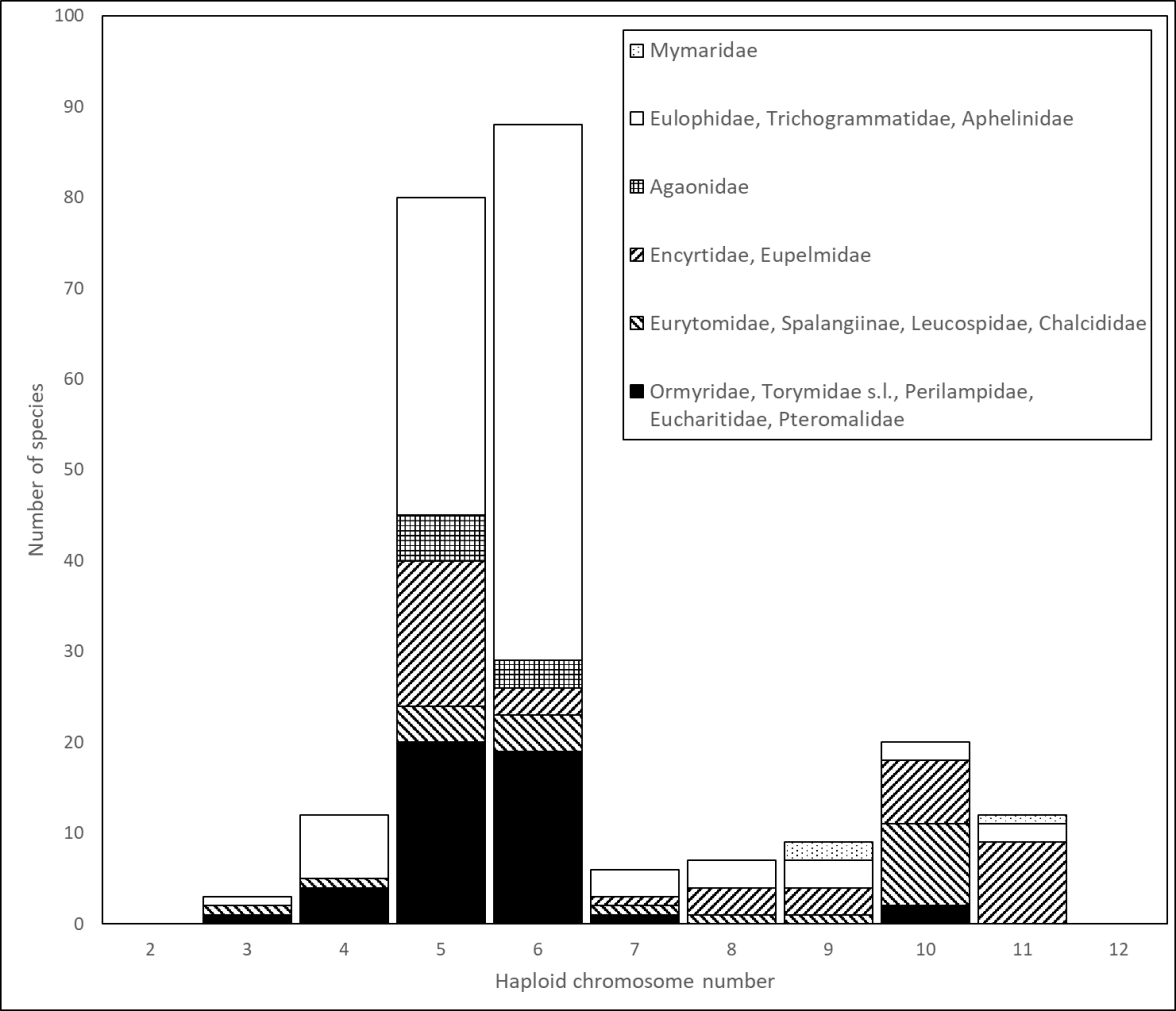
Distribution of main lineages of Chalcidoidea by the chromosome number at the species level (based on data from Table 1).

In the superfamily Chalcidoidea, haploid chromosome numbers (n) can vary from n = 3 to n = 11 (Table 1, Fig. 1). In fact, a few papers reporting n values outside of this range were also published during the previous century (Silvestri 1914; Muramoto 1993), but those results still need to be confirmed. Among chalcids, the distribution of chromosome numbers at the species level has a clear mode at n = 6, with a second local maximum at n = 10 (Fig. 1). Members of this superfamily with n = 5 are also very numerous, and the proportion of Chalcidoidea with other chromosome numbers is substantially smaller (Table 1, Fig. 1).

**Figure 2.**
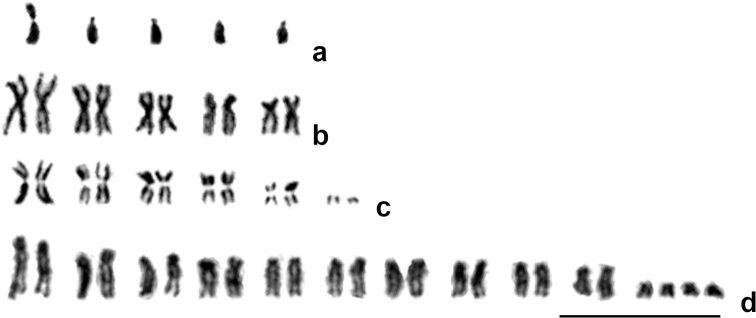
Representative karyotypes of Chalcidoidea**a***Trichogrammaprincipium* Sugonjaev & Sorokina, 1976 (Trichogrammatidae; n = 5) **b***Mesopolobusmediterraneus* (Mayr, 1903) (Pteromalidae; 2n = 10) **c***Oomyzusgallerucae* (Fonscolombe, 1832) (Eulophidae; 2n = 12) **d***Eurytomacynipsea* Boheman, 1836 (Eurytomidae; 2n = 20 + 4B). Scale bar: 10 µm.

**Figure 3.**
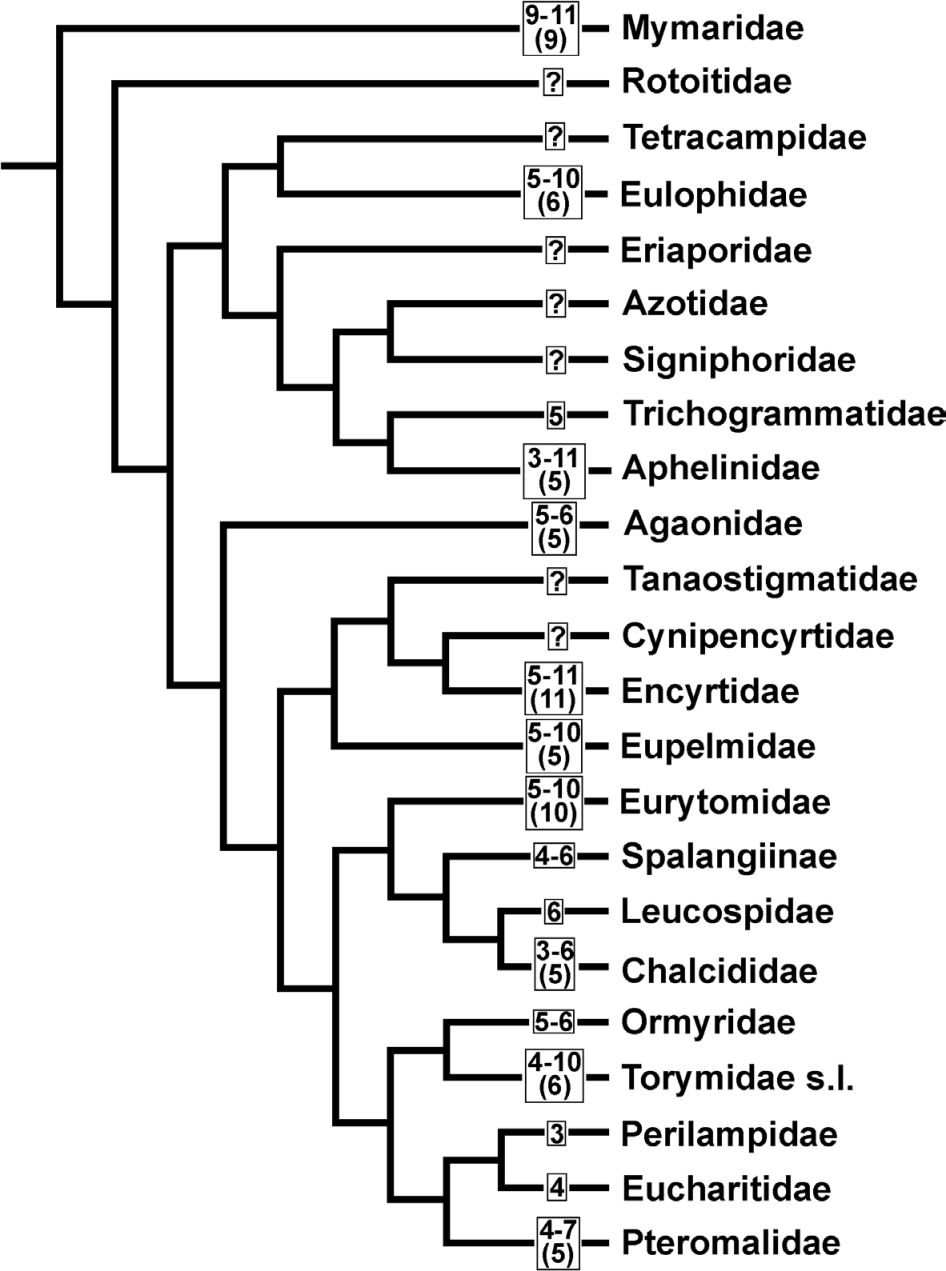
Variation ranges of chromosome numbers of Chalcidoidea mapped on the phylogenetic tree of chalcid families (simplified from Heraty et al. 2013). Most frequent chromosome numbers for certain taxa are given in brackets (redrawn from Gokhman 2013 and updated with data from Table 1).

Just a decade ago (Gokhman 2009; Gokhman and Gumovsky 2009), chalcid families were generally subdivided into two groups according to their chromosome numbers, i.e. the so-called “low-numbered” and “high-numbered” families. Within these groups, n values ranged from 3 to 7 and 8 to 11 respectively, with just a few exceptions. Most families belonged to the first group (Fig. 2a–c), whereas higher chromosome numbers were characteristic of Mymaridae, Eurytomidae, and Encyrtidae (Table 1, Fig. 2d). In addition, Aphelinidae contained taxa with both lower and higher n values. Specifically, the subfamily Aphelininae harbored parasitoids with n = 4–5, whereas Coccophaginae often had n = 10–11 (Gokhman 2009). However, n = 3–10 was found in different species of the large genus *Encarsia* Förster, 1878 from the latter subfamily (Baldanza et al. 1999). Moreover, n = 10 was detected in *Podagrionpachymerum* (Walker, 1833) and *P.gibbum* Bernard, 1938 (Torymidae) (Fusu 2008a). Furthermore, the above-mentioned pattern also substantially changed during the last years. For example, parasitoids with lower chromosome numbers (n = 5 to 7) were found within both Encyrtidae and Eurytomidae (Gokhman and Mikhailenko 2008; Gokhman 2010). These lower n values could be attributed to independent chromosomal fusions which took place in these groups. Finally, n = 8 to 10 were also detected in certain Eupelmidae and Eulophidae (Fusu 2008b, 2017; Gokhman and Nishkomaeva 2018). As a result of these findings, most principal lineages of Chalcidoidea now include both “high-numbered” and “low-numbered” members (Table 1, Fig. 3).

**Table 1.** Chromosome numbers of different families of Chalcidoidea. Spalangiinae were earlier considered as a subfamily of Pteromalidae s.l., but they deserve the family rank (Heraty et al. 2013). Torymidae s.l. include Megastigmidae (Janšta et al. 2018), but they are treated here as a single taxon because relationships of the latter group with other chalcid families remain uncertain. Data from: Fusu 2008b, 2009, 2017; Gokhman 2009, 2010, 2015b; Gokhman and Gumovsky 2009, 2013; Bolsheva et al. 2012; Gebiola et al. 2012; Gokhman et al. 2014b, 2017a, 2019b, c; Santos et al. 2015; Gokhman and Nishkomaeva 2018; Wu et al. 2019, the present paper and unpublished results of the author.

Family	No. species studied	Chromosome numbers (n)
Mymaridae	3	9, 11
Eulophidae	73	5, 6, 7, 8, 10
Trichogrammatidae	11	5
Aphelinidae	31	3, 4, 5, 6, 7, 8, 9, 10, 11
Agaonidae	8	5, 6
Encyrtidae	20	5, 8, 9, 10, 11
Eupelmidae	22	5, 6, 7, 8, 10
Eurytomidae	14	5, 6, 7, 8, 9, 10
Spalangiinae	2	4, 6
Leucospidae	1	6
Chalcididae	5	3, 5, 6
Ormyridae	2	5, 6
Torymidae s.l.	24	4, 5, 6, 10
Perilampidae	1	3
Eucharitidae	1	4
Pteromalidae	19	4, 5, 6, 7
**Total**	**237**	**3, 4, 5, 6, 7, 8, 9, 10, 11**

Chromosomes of Chalcidoidea are generally longer than those found in many other parasitoid Hymenoptera, mainly due to lower chromosome numbers that are characteristic of most chalcids, with average chromosome lengths ranging from 5 to 7 μm (Gokhman 2009). In this group, chromosomes of the “low-numbered” taxa mostly have two distinct arms, i.e. they are either metacentric or submetacentric (Gokhman 2013; Fig. 2b, c). Nevertheless, acrocentric and/or subtelocentric chromosomes can predominate as well, often within karyotypes of certain “high-numbered” chalcids (Gokhman and Gumovsky 2009; Fig. 2d, but see also Fig. 2a). Transitions from the latter character state to metacentrics/submetacentrics usually accompany the process of consecutive chromosomal fusions (see e g. Gokhman and Mikhailenko 2008).

Among Chalcidoidea, meiotic chromosomes were examined in some detail in a few dozen members of the families Eulophidae, Aphelinidae, Encyrtidae, Eupelmidae, Eurytomidae, Torymidae s.l. (including Megastigmidae) and Pteromalidae (Fusu 2009, 2017; Gokhman 2009 and references therein, Gokhman and Gumovsky 2013; Gokhman et al. 2014b). Specifically, chalcid chromosomes can form rod-like, cross-like or ring-like bivalents in diplotene, as in other members of the order Hymenoptera. Each bivalent usually carries one or two terminal/subterminal chiasmata.

### Chromosomal mutations

The following types of chromosomal mutations are characteristic of chalcid karyotypes: (Gokhman 2009): inversions, fusions (both central and tandem ones), translocations, polyploidy, aneuploidy and B chromosome variation. In addition, deletions/duplications probably also occur in this superfamily. Specifically, inversions were detected in certain members of the genus *Aphelinus* Dalman, 1820 (Aphelinidae) (Gokhman et al. 2017a). In this group, haploid karyotypes of most parasitoids that belong to the *varipes* species group with n = 4, contain two metacentric and two acrocentric chromosomes. However, *A.hordei* Kurdjumov, 1913 also has a similar karyotype structure, but the centromere of the second metacentric is significantly shifted towards the chromosome end, and in a certain sister species, *A.kurdjumovi* Mercet, 1930, this centromere becomes terminal, turning the particular chromosome into an acrocentric (Gokhman et al. 2017a). An inversion could also be involved in the process of karyotype transformation within the *Lariophagusdistinguendus* species complex (König et al. 2019).

At present, direct evidence for translocations, which occur among Chalcidoidea, is generally scarce. For instance, reciprocal translocations are presumed in certain members of the family Eulophidae (Gokhman 2009). These rearrangements, together with deletions and duplications, are apparently responsible for the numerous size differences between chromosomes of related chalcid species with the same n values (Giorgini and Baldanza 2004; Gebiola et al. 2012). Comparative studies of the genome size complemented with chromosome morphometrics can provide additional insights regarding possible deletions/duplications in closely related forms with similar karyotypes (Gokhman et al. 2017a). Nevertheless, detection of these mutations often requires sophisticated techniques of the chromosome study (see e.g. Gokhman et al. 2019a), and therefore more rearrangements of this kind are undoubtedly going to be discovered within chalcid karyotypes in the future.

Fortunately, other types of chromosomal mutations can be identified more easily among the Chalcidoidea, because these karyotypic changes usually affect the chromosome number of related forms. For example, this parameter decreases via chromosomal fusions, and the products of these rearrangements can be instantly detected using e.g. chromosome morphometrics or whole chromosome painting (Gokhman et al. 2019a; König et al. 2019). Specifically, more or less well-documented consecutive chromosomal fusions were found in the Eurytomidae. Although parasitoids that belong to this group, and to the genus *Eurytoma* in particular, generally have n = 10 (Fig. 2d), but n = 5, 6 and 7 were found in *E.compressa* (Fabricius, 1794), *E.serratulae* (Fabricius, 1798) and *E.robusta* Mayr, 1878 respectively (Gokhman and Mikhailenko 2008). The number of larger metacentrics observed in these chalcids also corresponded with the above-mentioned scenario. Analogously, two studied members of the genus *Sycophila* Walker, 1871 from the same family, namely, *S.submutica* (Thomson, 1876) and *S.biguttata* (Swederus, 1795), have n = 8 and 9 respectively (Gokhman and Mikhailenko 2008; Gokhman and Gumovsky 2013). Furthermore, n = 10 is characteristic of both *Metaphycusflavus* (Howard, 1881) and *M.luteolus* (Timberlake, 1916) (Encyrtidae), but n = 9 and 5 were respectively found in *M.angustifrons* Compere, 1957 and *M.stanleyi* Compere, 1940 (Gokhman 2010). In addition, Gokhman et al. (2019a) who applied chromosome microdissection and whole chromosome painting to chromosome sets of two cryptic species of *Lariophagusdistinguendus* complex with n = 5 and 6, were able to identify chromosomes involved in a particular fusion. During this process, the only acrocentric and a medium-sized metacentric in the chromosome set with n = 6 fused into the largest metacentric chromosome in the karyotype with n = 5. At present, however, it is difficult to distinguish between centric and tandem fusions in the superfamily Chalcidoidea. Nevertheless, since the haploid chromosome set containing eleven subtelocentrics or acrocentrics of similar size is considered ancestral for chalcids (Gokhman 2013), centric fusions could predominate in this group.

Polyploid individuals were found in a few groups of Chalcidoidea. For example, triploid females were found in *Nasoniavitripennis* and certain Aphelinidae (Gokhman 2009 and references therein). In the former species, diploid males and tetraploid females were also detected. However, various attempts to create a stable strain of *N.vitripennis* with tetraploid females and diploid males failed, probably due to the so-called preferential segregation of chromosomes (Crozier 1975). Nevertheless, a particular stock of *N.vitripennis* with triploid females/diploid males can be supported in the lab for many generations (Leung et al. 2019).

At present, the only reliable case of aneuploidy among chalcids is known in *Torymusbedeguaris* (Linnaeus, 1758) (Torymidae). In this species, which usually has 2n = 12, three copies of the smallest acrocentric chromosome carrying NORs were found in the only specimen with 2n = 13 (Gokhman et al. 2014a). In addition, Baldanza et al. (1999) reported n = 11 in a few male individuals of *Encarsiaasterobemisiae* Viggiani & Mazzone, 1980 (Aphelinidae) normally having n = 10 and 2n = 20. However, this pattern was apparently caused by presence of a particular B chromosome (see below).

Up to now, B chromosomes were found in certain members of the superfamily Chalcidoidea. Specifically, the so-called PSR (paternal sex ratio) B chromosomes were detected in two distantly related chalcid species, i.e. *Nasoniavitripennis* and *Trichogrammakaykai* (Nur et al. 1988; Van Vugt et al. 2005). These paternally inherited chromosomes eliminate all other elements of the paternal genome from the diploid zygote, thus turning it into the haploid one. In addition, B chromosomes which apparently do not carry sex-ratio distorting factors, were also found in a few members of the families Aphelinidae and Eulophidae (Baldanza et al. 1999; Gebiola et al. 2012; Gokhman et al. 2014b). For example, the highest number of B chromosomes among parasitoids was detected in *Pnigaliogyamiensis* Myartseva & Kurashev, 1990 (Eulophidae) with 2n = 12 + 0–6B (Gokhman et al. 2014b). Chromosomes of this kind have also been recently found in *Eurytomacynipsea* Boheman, 1836 with 2n = 20 + 0–4B (Fig. 2d).

### Phylogenetic implications of chromosomal characters

Chalcid karyotype evolution was previously studied using phylogenetic reconstructions that were based on morphological and/or molecular characters (Gokhman 2009, 2013, see also Gokhman and Gumovsky 2009). Together with other papers published during the last 10–15 years (Gokhman and Mikhailenko 2008; Gokhman 2010; Santos et al. 2015; Gokhman et al. 2017a), these studies revealed a number of synapomorphies of certain higher taxa (e.g. lower chromosome numbers shared by the Eucharitidae and Perilampidae, see Fig. 3) and related species. The best known synapomorphies of the latter kind are represented either by chromosomal fusions in the Eurytomidae and Encyrtidae or by inversions in the Aphelinidae (see above). However, understanding karyotype evolution of many supraspecific taxa of parasitic wasps is far from straightforward. For instance, a detailed molecular analysis suggests n = 6 as an ancestral chromosome number for the *Lariophagusdistinguendus* complex (König et al. 2019), although n = 5 is currently considered as an ancestral value for the family Pteromalidae in general (Gokhman 2009).

The problem of phylogenetic reconstruction of karyotype evolution at the level of higher taxa can be illustrated by the example of the Eulophidae, apparently the best studied group of the superfamily Chalcidoidea (Table 1). Indeed, the haploid chromosome set containing five larger metacentrics and a smaller subtelocentric/acrocentric (n = 6) was long considered ancestral for the family, since it predominates in most previously examined lineages of Eulophidae (Gokhman 2009 and references therein). In that case, the karyotype of *Trichospilusdiatraeae* which contains four longer metacentric and three shorter acrocentric chromosomes (n = 7), might originate from a centric fission from the apparently ancestral chromosome set (Gokhman et al. 2017b). However, a recent study of *Ophelimusmaskelli* (Ashmead, 1900), the only member of the subfamily Opheliminae with the known karyotype, revealed n = 10 (Gokhman and Nishkomaeva 2018). Since this subfamily apparently represents a less derived group of Eulophidae (see e.g. Gumovsky 2008), n = 10 is likely to be considered ancestral for the family in general, with n = 7 and 6 arose from the preceding karyotype by consecutive chromosomal fusions (Gokhman and Nishkomaeva 2018).

In addition, numerous chromosomal fusions lead to independent origins of similar karyotypes within different lineages of Chalcidoidea (Gokhman 2013). Specifically, at least some chromosome sets with n = 10 originated from the apparently ancestral karyotype containing eleven subtelocentrics/acrocentrics through pairwise fusions. Moreover, further consecutive rearrangements of this kind also led to the multiple origins of chalcid chromosome sets with n = 6 (five larger metacentrics/submetacentrics and a smaller subtelocentric/acrocentric; Fig. 2c). In turn, numerous karyotypes with five metacentric chromosomes (n = 5; Fig. 2b) also can originate through independent fusions of the above-mentioned subtelocentrics/acrocentrics to certain metacentric chromosomes (Gokhman 2013). These parallel transitions apparently occurred in a few distantly related chalcid families, including Eulophidae, Agaonidae, and Torymidae s.l. plus Ormyridae (Fig. 3).

### Taxonomic implications of chromosomal characters

In the superfamily Chalcidoidea, karyotypic features can have substantial taxonomic implications, and these implications are the most important at the species level (Gokhman 2015b). Specifically, in a few cases different karyotypes were reported for the same parasitoids. Although some of those reports apparently resulted from misidentifications of well-defined different species (see Gokhman 2009 and references therein), cryptic taxa were also involved in certain cases. For example, a chromosome study of the supposedly well-known synanthropic parasitoid of many stored-product pests, *Anisopteromaluscalandrae* (Howard, 1881) (Pteromalidae), eventually resulted in the detection and description of a new cosmopolitan species, *A.quinarius* Gokhman & Baur, 2014, with these species respectively having n = 7 and 5 (Baur et al. 2014). Analogously, two morphologically indistinguishable cryptic species with n = 5 and 6 were found in the *Lariophagusdistinguendus* complex from the same family (König et al. 2019). In addition, two newly described members of the genus *Eupelmus* Dalman, 1820 (Eupelmidae), *E.barai* Fusu, 2017 and *E.vladimiri* Fusu, 2017, were earlier misidentified as *E.vesicularis* (Retzius, 1783) and *E.impennis* Nikol’skaya, 1952, although the first, the last, and the two remaining species have n = 6, 9, and 5 respectively (Fusu 2017). Similar cases are summarized and discussed in the recent review on integrative taxonomy of parasitoid Hymenoptera (Gokhman 2018).

Variation of chromosome morphology between routinely stained karyotypes of related species with the same n values was also revealed. For instance, two reproductively isolated populations of *Encarsiasophia* (Girault & Dodd, 1915) (Aphelinidae) from Spain and Pakistan have structurally different karyotypes with n = 5 (Giorgini and Baldanza 2004). We also found that chromosome sets of two members of the genus *Trichogramma* Westwood, 1833 with n = 5, i.e. *T.pretiosum* Riley, 1879 and *T.principium* Sugonjaev & Sorokina, 1976, substantially differ in their morphometric parameters (Gokhman et al. 2017b and the present paper; Fig. 2a), contrary to some previous reports for this genus (Hung 1982). Up to now, various techniques of differential staining did not reveal karyotypic differences between closely related species with the same morphology of chromosomes, but this seems possible, given the fact that members of the same genus, for instance, can differ in the number and localization of NORs (Baldanza and Giorgini 2001; Giorgini and Baldanza 2004; Gokhman et al. 2014a).

## Future directions

In the coming decades, karyotypic study is undoubtedly going to become an important tool of taxonomic and cytogenetic research on many groups of parasitic wasps, including chalcids. However, this investigation can be effective only if complemented by other modern approaches and techniques. For example, it should be used in combination with a thorough morphological analysis for detecting and identifying cryptic species of parasitoids (Gokhman 2018). This is especially true for the families with a relatively high variation in chromosomal characters, e.g. Encyrtidae, Aphelinidae, Eurytomidae, Pteromalidae etc. (Gokhman 2015a). Since the genome size is generally correlated with the total length of chromosomes, but not necessarily with the overall karyotype structure (Gokhman et al. 2017a), a combined study can highlight hidden chromosomal rearrangements among closely related forms (see e.g. Moura et al. 2020). On the other hand, cytogenetic research of the superfamily Chalcidoidea per se will also benefit from using molecular and similar approaches, which include microdissection and chromosome painting (Gokhman et al. 2019a), immunochemical techniques (Bolsheva et al. 2012) and other applications. In turn, some of these techniques could be used to investigate fine structure of meiotic chromosomes of hybrids between closely related chalcid species (see e.g. König et al. 2019). Finally, modern efforts for genome sequencing can also be supported by cytogenetic studies of the Chalcidoidea in a number of ways – from providing direct estimates of the number of linkage groups (which equals to the n value) to the physical mapping of various DNA sequences, especially repetitive ones, using FISH (Gokhman 2009; Gokhman et al. 2017a).

## Conclusion

Although a considerable amount of new data of the karyotypic study of the superfamily Chalcidoidea were collected and summarized during the last decade (see e.g. Gokhman 2015a), chromosomes of many chalcid taxa remain totally unknown. Nevertheless, conclusions based on the accumulated data already have important implications for genetics, taxonomy and phylogeny of this enormous group, as well as for its use in biological pest control (Gokhman 2015b, 2018). In turn, phylogenetic and taxonomic research provides essential information which enables better understanding of various cytogenetic phenomena occurring in the Chalcidoidea (Baur et al. 2014; Fusu 2017; Gokhman et al. 2017a; König et al. 2019), and I am sure both these trends are certainly going to continue in the observable future.
